# Design principles for robust multistability in coupled feedforward-feedback regulatory circuits

**DOI:** 10.1038/s41540-026-00731-1

**Published:** 2026-05-02

**Authors:** Hong Qi, Zhi-Yu Zhao, Yu-Song Yin, Xiang Li, Chen Jia, Lei Zhang

**Affiliations:** 1 Complex Systems Research Center, Shanxi University, Taiyuan, China; 2 Shanxi Key Laboratory for Mathematical Techniques in Complex Systems, Shanxi University, Taiyuan, China; 3 Key Laboratory of Complex Systems and Data Science of Ministry of Education, Shanxi University, Taiyuan, China; 4 Department of Physics and Fujian Provincial Key Laboratory for Soft Functional Materials Research, Xiamen University, Xiamen, China; 5 Applied and Computational Mathematics Division, Beijing Computational Science Research Center, Beijing, China; 6 Beijing International Center for Mathematical Research, Peking University, Beijing, China; 7 Center for Quantitative Biology, Peking University, Beijing, China; 8 Center for Machine Learning Research, Peking University, Beijing, China

**Keywords:** Engineering, Mathematics and computing, Physics

## Abstract

Feedforward loops (FFLs) and feedback loops (FBLs) are ubiquitous network motifs that mediate signal filtering, pulse generation, and state switching; yet, how coupling FBLs to FFLs produces robust multistability—a key mechanism for cellular decision-making—remains unclear. Here, we systematically investigate coupled FFL–FBL architectures by focusing on two prevalent FFL types, each with AND or OR logic, yielding four distinct frameworks. For each framework, we enumerate all 3^6^ = 729 possible circuits, corresponding to three possible states (activation, inhibition, or absence) for each of six feedback edges, formulate each circuit as a system of ordinary differential equations, and quantify robustness as the proportion of 100,000 randomly sampled parameter sets exhibiting multistability. Our results reveal two key principles. First, positive self-activation is a primary driver of multistability, but the identity of the critical node(s) depends on the FFL type and logic. Second, coherent FFLs support multistability more readily than incoherent ones, whereas the choice between AND and OR logic has a comparatively weaker effect. Notably, we identify representative high-performing circuits within each framework and find that a small set of circuit designs remain robustly multistable across all four frameworks. These findings advance the theoretical understanding of motif design and provide practical guidelines for engineering synthetic multistable circuits.

## Introduction

Complex networks, especially biological networks, are often composed of a small set of recurring regulatory patterns known as network motifs^1^. These motifs serve as basic building blocks of networks, each capable of performing specific information-processing functions^2^. Among these motifs, feedforward loop (FFL) and feedback loop (FBL) are the most extensively studied^2–8^. The canonical topologies of these motifs are illustrated in Supplementary Fig. S1. In general, FFLs propagate information from input to output through parallel direct and indirect paths, whereas FBLs feed information back to the input.

The simplest FFL consists of three nodes, denoted as X, Y, and Z. In this configuration, X acts as the input, Z as the output, and Y as an intermediate regulator. The loop comprises two paths: a single-edge direct path from X to Z and a two-edge indirect path from X through Y to Z. Each of the three edges can be either activating or repressing, giving 2^3^ = 8 possible FFL types. The first four are coherent FFLs, where the effect of the direct path is consistent with the overall effect of the indirect path. The remaining four are incoherent FFLs, where the direct effect contradicts the indirect effect^2^. Their distribution in biological networks is highly uneven: the coherent type-1 FFL (C1-FFL, where X activates Y and Z, and Y activates Z) and the incoherent type-1 FFL (I1-FFL, where X activates both Y and Z, but Y represses Z) are far more prevalent than the other six^9,10^. Additionally, the other six exhibit reduced functionality compared with these two dominant types^11^. Therefore, our study focuses exclusively on C1-FFL and I1-FFL. To fully describe the dynamics of an FFL, one must also specify the regulatory logic that integrates the effects of X and Y on Z, namely an AND gate (both inputs required) or an OR gate (either input sufficient)^11^.

An FBL is a closed circuit in which the output feeds back to its input, allowing the system to self-regulate^6^. Negative FBLs are essential for maintaining stable oscillations, whereas positive FBLs are required for achieving multistability^12–14^. Multistability—the ability of a system to occupy multiple stable internal states under the same external conditions—has a pivotal impact on the functions of such systems^15,16^. While bistability, as a simple and representative instance of multistability, has long been the focus of extensive research^17–19^, more complex forms such as tristability^20–22^, quadrastability^23–25^, and even septastability^26^ have only recently attracted significant attention.

FFLs and FBLs are prevalent as individual motifs in biological networks, and substantial experimental evidence demonstrates that they frequently appear together^27–34^. Representative coupled architectures include: (i) In the apoptotic switch controlled by the Bcl-2 family, Bim initiates apoptosis by directly activating Bax and indirectly inhibiting Bcl-2, thereby preventing it from restraining Bax; activated Bax further activates inactive Bax and forms pores, leading to mitochondrial outer membrane permeabilization (MOMP)^35–37^. (ii) Following MOMP, XIAP inhibits Casp9 and Casp3, with Casp9 activating Casp3; Casp3-mediated cleavage of Casp9 creates a positive FBL that amplifies the death signal^32,38,39^. (iii) In PINK1-driven mitophagy, PINK1 activates Parkin directly via phosphorylation of Parkin and indirectly through phosphorylation of ubiquitin^33,40^; Parkin, in turn, facilitates the accumulation of ubiquitin chains, which are then phosphorylated by PINK1, creating a positive FBL that amplifies mitophagy signals^41,42^. (iv) For mitochondrial permeability transition pore (PTP) opening, Ca^2+^ entry promotes reactive oxygen species (ROS) generation, and together they trigger pore opening^43,44^; ROS enhances Ca^2+^ influx and induces further ROS generation, yielding two positive FBLs^45,46^.

Despite their diverse biological contexts, these examples share two common features. First, each system is organized around an FFL, supplemented by one or more FBLs. Second, their dynamics exhibit multistability. Bcl-2 family-regulated MOMP^31^, XIAP-mediated apoptosis^38^, and PINK1-driven mitophagy^47^ all display bistability, meaning they can reside in two stable states and switch between them in response to specific signals. A more complex scenario is PTP opening, which operates as a tristable switch and provides a more subtle mechanism for controlling mitochondrial permeability^34^. Moreover, by combining experimental measurements with mathematical modeling, Yao et al. identified a minimal circuit in which an FFL is coupled to a positive FBL, producing robust, resettable bistability that underlies mammalian cell-cycle entry^48^. Using a similar integrative approach, Lu et al. demonstrated that a core circuit composed of miR-200 and ZEB, mutually inhibitory and both activated by SNAIL (interpretable as a coupled FFL–FBL structure), functions as a ternary switch regulating epithelial–mesenchymal cell fate^49^.

It is well known that the C1-FFL acts as a persistence filter, whereas the I1-FFL functions as a pulse generator and response accelerator^2^. However, the biological examples discussed above show that cells often require coupled architectures in which an FFL provides the core framework, supplemented by FBLs, to achieve multistability. This naturally raises the question of which types of coupled FFL-FBL structures can robustly generate multistability, and further, what decisive factors govern this behavior. To address this question, we systematically investigate two canonical FFL types (C1 and I1), each implemented with AND and OR logic, resulting in four distinct FFL frameworks. By enumerating all 729 possible three-node circuits per framework and performing large-scale parameter screening and representative bifurcation analyses, we identify two key principles: (i) positive self-activation emerges as a primary mechanism promoting multistability, but the critical node(s) depends on FFL type and logic; and (ii) coherent FFLs generally support multistability more readily than incoherent ones, though a small set of topologies are robust across frameworks. These results not only deepen theoretical understanding of motif-level design principles but also provide a concise, generalizable blueprint for designing multistable regulatory circuits with potential applications in synthetic biology, signal processing, and cellular decision-making.

## Results

### Circuit enumeration and multistable robustness assessment

**Fig. 1:**
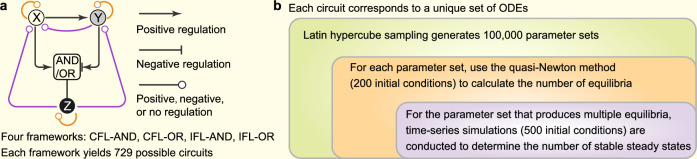
Overview of circuit frameworks and workflow for identifying multistable robustness. **a** Schematic representation of the four circuit frameworks. Each circuit comprises three nodes: an input (X), an intermediate (Y), and an output (Z), forming a feedforward loop (FFL) that can be either coherent or incoherent. Z is regulated by a logic gate (AND/OR). Accordingly, four frameworks are defined: CFL-AND (coherent FFL with AND logic), CFL-OR (coherent FFL with OR logic), IFL-AND (incoherent FFL with AND logic), and IFL-OR (incoherent FFL with OR logic). Feedback regulation among these nodes is indicated by six colored edges, each of which can be positive, negative, or absent. Considering all possible feedback combinations, each framework encompasses 729 distinct circuits. **b** Workflow for assessing multistability and robustness. Each circuit is translated into a set of ordinary differential equations (ODEs), with 100,000 parameter sets generated via Latin hypercube sampling. Equilibrium points are computed using the quasi-Newton method with 200 initial conditions per parameter set. For parameter sets exhibiting multiple equilibria, time-series simulations with 500 initial conditions determine the number of stable steady states. This workflow systematically evaluates the multistable robustness of each circuit.

Our goal is to investigate the multistable behavior of a coupled architecture centered around an FFL and supplemented by several FBLs. Since C1-FFL and I1-FFL comprise over 80% of all FFLs, we focus on these two types, employing AND and OR gates, respectively. Accordingly, this study encompasses four distinct frameworks: C1-FFL with an AND gate (CFL-AND), C1-FFL with an OR gate (CFL-OR), I1-FFL with an AND gate (IFL-AND), and I1-FFL with an OR gate (IFL-OR). To identify network configurations capable of generating multistability, we examine all possible circuits of three nodes: one that receives input (X), a second that transmits output (Z), and a third (Y) that connects them and forms a logic gate with X to regulate Z. As illustrated in Fig. 1a, each framework (CFL-AND, CFL-OR, IFL-AND, IFL-OR) delineates a specific type of FFL, where six possible feedback edges may connect nodes to themselves or to other nodes, and each of these edges can represent positive, negative, or no regulation, resulting in 3^6^ = 729 possible circuits per framework.

An overview of the scheme for searching multistable circuits and assessing their robustness is outlined in Fig. 1b. First, each circuit corresponds to a unique set of ordinary differential equations (ODEs). We treat each node in the circuit as a transcription component, which exists in both active and inactive forms. A single circuit translates into a system of rate equations where the regulatory interactions among the three components are described using Hill functions. Moreover, basal activation and inactivation are included for each component. Second, each set of ODEs is assigned to 100,000 parameter sets generated by the Latin hypercube sampling method within a predefined parameter space which is considered to be biologically feasible. These random parameter sets served as surrogates of diverse cellular activities across various cell types and cellular environments^48^. Third, for each parameter set, we employ a quasi-Newton method with a Jacobian-free implementation (see Supplementary Note 1 for algorithmic details) to compute all equilibrium points (both stable and unstable) of each corresponding set of ODEs. To assess whether the dynamical system has multiple equilibrium points, 200 initial values are selected for each parameter set, allowing us to explore the system’s behavior under varying initial conditions. Consequently, we analyze a total of 14.58 billion dynamical systems (729 × 100,000 × 200) using the quasi-Newton method for each framework. Fourth, if a parameter set yields more than two equilibrium points, we numerically compute the time evolution with 500 initial conditions to verify the presence of multistability and subsequently determine the number of stable steady states. Finally, we define the *Robustness Percentage* of a circuit as the fraction of the 100,000 sampled parameter sets (generated by Latin hypercube sampling within the predefined parameter space) that produce multistability. For each parameter set that enables multistability in the circuit, we also record the corresponding type of multistability, i.e., bistability, tristability, quadrastability, or pentastability.

### Classification of all 729 circuits across four frameworks by multistable robustness

For each circuit within the four frameworks, we employ the same quasi-Newton framework with a multi-initialization and solution-filtering strategy (described in Supplementary Note 1) to evaluate its *Robustness Percentage*. Under the predefined biologically feasible parameter space and the Latin hypercube sampling scheme described above, the *Robustness Percentages* of all circuits fall within a relatively small range (0–5%).

Based on these *Robustness Percentage*, we use the k-means clustering algorithm to classify the 729 circuits of each framework into five categories: high, medium high, medium, medium low, and low. Because the classification is performed using k-means clustering on the obtained robustness values, the category boundaries are determined in a data-driven manner rather than by predefined thresholds. These details, primarily the Robustness Percentages of the 729 circuits in each framework and their corresponding classifications, together with supplementary information on circuit IDs and the properties of their six feedback edges (−1, 0, and 1, representing inhibition, no regulation, and activation, respectively), are provided in Supplementary Data.

**Fig. 2:**
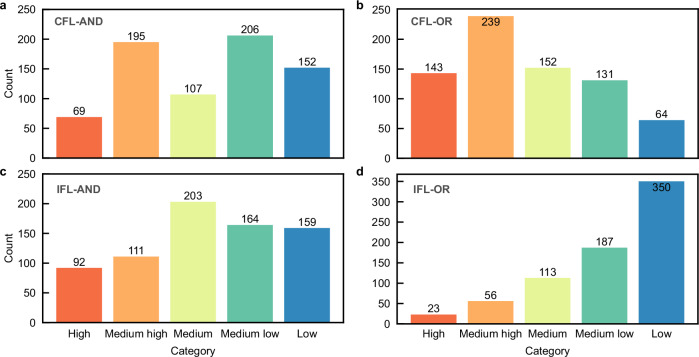
Distribution of circuits across five robustness categories for four frameworks. Each framework consists of 729 circuits, which are classified into five categories based on their *Robustness Percentages*: high, medium high, medium, medium low, and low. The subfigures represent the four frameworks: CFL-AND (**a**), CFL-OR (**b**), IFL-AND (**c**), and IFL-OR (**d**).

Figure 2 illustrates the distribution of the 729 circuits across the five robustness categories for each of the four frameworks: CFL-AND, CFL-OR, IFL-AND, and IFL-OR. CFL-AND exhibits a balanced distribution, with the highest count (206 circuits) in the medium low category and the lowest count (69 circuits) in the high category. CFL-OR shows a prominent concentration in the medium high category (239 circuits), while the low category contains the fewest circuits (64). IFL-AND is predominantly distributed in the medium category (203 circuits), with the high category accounting for only 92 circuits. In contrast, IFL-OR is heavily skewed toward the low category, which contains 350 circuits, whereas the high category includes only 23 circuits. Overall, in most frameworks the high category contains the fewest circuits, except for CFL-OR, where the low category has the fewest circuits. Conversely, the low category has the highest number of circuits in IFL-OR.

### Edge-level determinants of multistable robustness across categories

**Fig. 3:**
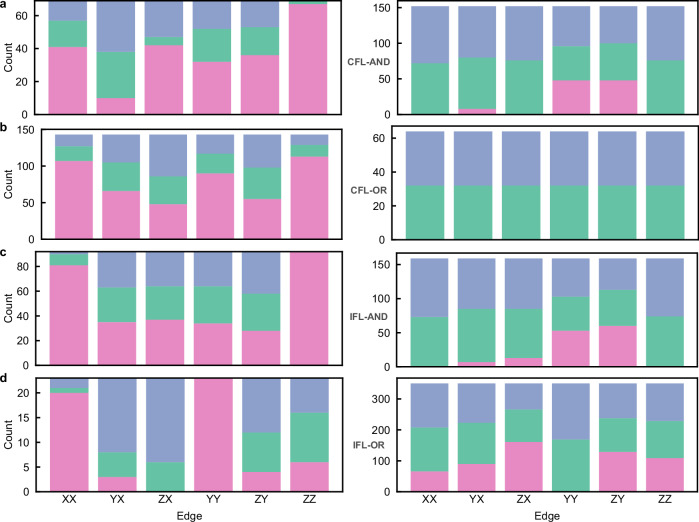
Distribution of feedback regulation types in the high and low categories across four frameworks. The frameworks shown are CFL-AND (**a**), CFL-OR (**b**), IFL-AND (**c**), and IFL-OR (**d**). The left panels represent the high category, and the right panels represent the low category. Each panel displays the distribution of three regulatory effects—positive (pink), none (green), and negative (blue)—for six feedback edges (XX, YX, ZX, YY, ZY, ZZ).

There are six feedback edges, each representing a specific regulatory effect: XX denotes the effect of X on itself, YX the effect of Y on X, ZX the effect of Z on X, YY the effect of Y on itself, ZY the effect of Z on Y, and ZZ the effect of Z on itself. Figure 3 compares these feedback edges in the high and low categories across the four frameworks (CFL-AND, CFL-OR, IFL-AND, and IFL-OR) based on their regulatory effects: positive, none, and negative. The goal is to identify which feedback edges serve as key determinants distinguishing circuits in the high category from those in the low category, following two principles: (1) If the three effects of an edge are evenly distributed within either the high or low category, the edge is unlikely to be a significant factor for classification; (2) If the distributions of effects differ markedly between the high and low categories, the edge is likely a key determinant.

For CFL-AND, ZZ emerges as the critical determinant, as it is almost entirely positive in the high category but exclusively none or negative in the low category. In CFL-OR, the classification is more complex. While no edge in the low category exhibits a positive effect, all six edges in the high category include positive, none, and negative effects. Among these, XX, YY, and ZZ display a higher proportion of positive effects in the high category, indicating that they are likely key factors. For IFL-AND, the results are relatively straightforward. ZZ is entirely positive in the high category but exclusively none or negative in the low category. Additionally, XX shows predominantly positive effects in the high category, while no positive effects are observed in the low category. This suggests that XX and ZZ are the primary factors distinguishing the high and low categories. Finally, for IFL-OR, the distinction is the clearest: YY stands out among the six edges, as it is entirely positive in the high category while exclusively none or negative in the low category, which establishes it as the key determinant for classification in this framework.

To complement the visual inspection in Fig. 3, we perform a statistical association analysis (Supplementary Table S1). For each framework and each feedback edge, contingency tables are constructed to examine the association between regulatory effect (positive/none/negative) and robustness category (high/low). Independence between the two variables is assessed using *χ*² tests, and the strength of association is quantified using Cramer’s *V*. Because the large number of circuits leads to widespread statistical significance, edge importance is interpreted primarily according to Cramer’s *V* rather than *p*-value. The statistical analysis is largely consistent with the visual inspection. The same key edges are identified across all frameworks, except that in CFL-AND two additional edges (ZX and XX) exhibit non-negligible association. Nevertheless, ZZ shows the largest Cramer’s *V* by a clear margin within this framework.

**Fig. 4:**
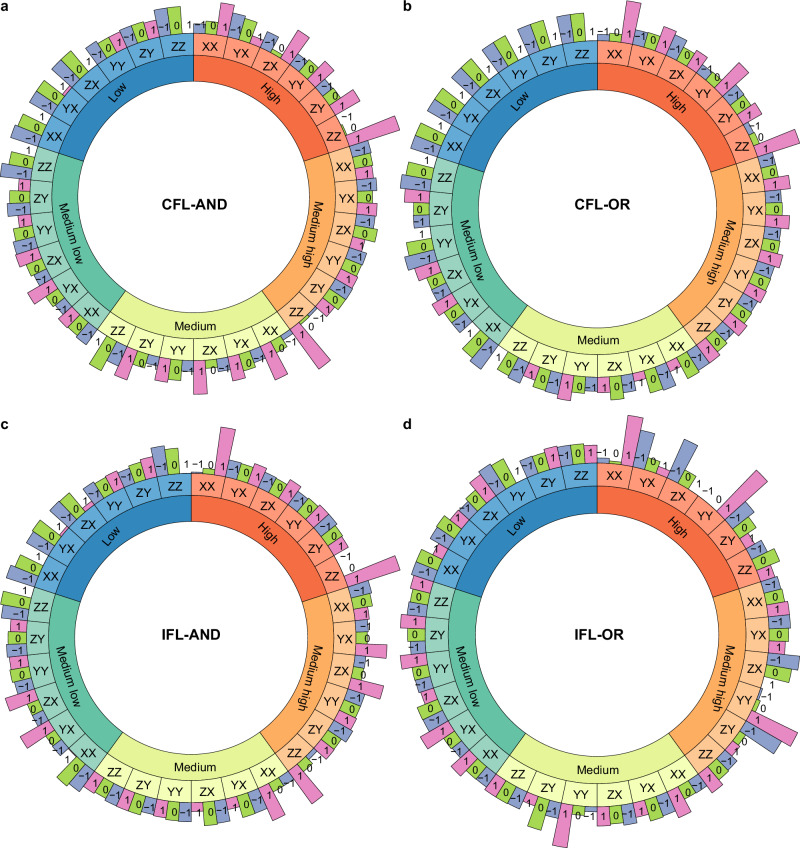
Proportions of regulatory effects across six feedback edges in five categories for four frameworks. The frameworks shown are CFL-AND (**a**), CFL-OR (**b**), IFL-AND (**c**), and IFL-OR (**d**). The sunburst plots represent the five categories (high, medium high, medium, medium low, and low) in the inner layer, the six feedback edges (XX, YX, ZX, YY, ZY, ZZ) for each category in the middle layer, and the distribution of the three effects for each feedback edge in the outer layer: positive (1), no effect (0), and negative (–1).

We next identify the key determinants for multistability in each framework by comparing the proportions of the three types of regulatory effects (positive, none, and negative) for the six feedback edges across the five categories. For each sunburst plot in Fig. 4, the inner layer represents the five categories (high, medium high, medium, medium low, and low), the middle layer shows the six feedback edges (XX, YX, ZX, YY, ZY, ZZ) for each category, and the outer layer depicts the distribution of the three effects for each feedback edge.

Figure 4a highlights that the positive effect of ZZ is predominantly observed in the high and medium high categories, with no such effect in the other three categories. Thus, for CFL-AND, the self-activation of Z is the key factor driving multistability. Figure 4b displays that the positive effects of XX, YY, and ZZ are notably prominent in the high category but absent in the medium low and low categories. Therefore, the self-activation of X, Y, and Z emerges as the key factors for multistability in CFL-OR. Figure 4c reveals that XX’s positive effect is more evident in the high category, while ZZ’s positive effect is stronger in both the high and medium high categories, with both effects being nearly absent in the medium low and low categories. Hence, the self-activation of X and Z plays a crucial role in IFL-AND. Finally, Fig. 4d indicates that YY displays a positive effect exclusively in the high category and is entirely absent in the low category, while all three types of effects are present in the remaining categories. Thus, for IFL-OR, the self-activation of Y is the key determinant for multistability.

### Global circuit atlas and key origins of robust multistability

**Fig. 5:**
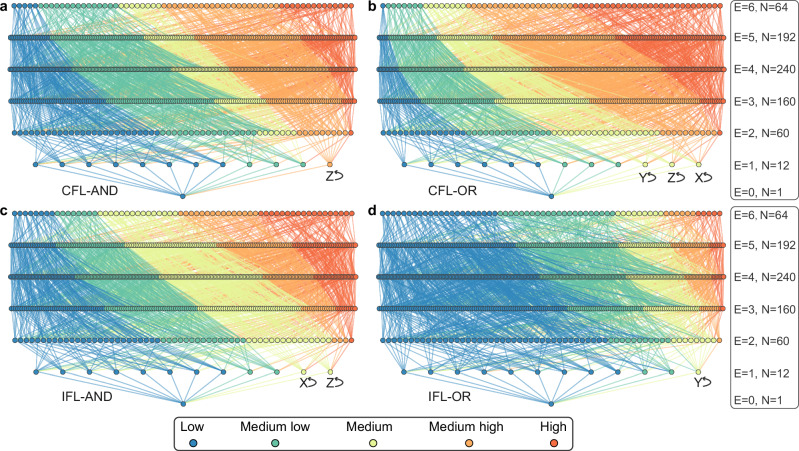
Comprehensive atlas of 729 circuits for four frameworks. The frameworks shown are CFL-AND (**a**), CFL-OR (**b**), IFL-AND (**c**), and IFL-OR (**d**). For each framework, one node represents one circuit. All circuits are arranged from bottom to top based on their topological complexity, represented by the number of edges, denoted as E, and within each row, they are color-sorted by their *Robustness Percentages* from left to right, with each row containing N circuits. Any two circuits with a one-edge difference are connected, and the color of the connection is determined by the color of the upper-layer circuit. The circuit labeled with X, Y, and Z indicates that circuits with high *Robustness Percentages* are derived from this circuit or combinations of these circuits.

Figure 5 illustrates the global relationships among 729 circuits within each framework, further identifying the key determinants of multistability. Each subfigure is organized hierarchically, with circuits increasing in the number of feedback edges (E) from bottom to top. For example, the lowest layer represents circuits with no feedback edges (containing only feedforward loops), while the topmost layer includes circuits with all six feedback edges. The number of circuits (N) varies across layers, as indicated in the legend. Within each layer, circuits are arranged from left to right in ascending order of *Robustness Percentages*, highlighting the diversity in robustness among circuits with the same topological complexity.

This comprehensive atlas, similar to those described in previous studies^25,50^, connects circuits such that one can transition into another by adding or removing a single edge. Such connectivity facilitates the identification of circuits with high *Robustness Percentages* and their origins from basic circuits containing only a single feedback edge. Specifically, the high-robustness circuits in CFL-AND originate from the self-activation of Z (Fig. 5a), those in CFL-OR from the self-activation of X, Y, and Z (Fig. 5b), those in IFL-AND from the self-activation of X and Z (Fig. 5c), and those in IFL-OR from the self-activation of Y (Fig. 5d). These results reinforce the conclusion that self-activation of specific feedback edges serves as the key determinant of robust multistability, consistent with the edge-level analysis in Fig. 4.

### Representative circuit analysis reveals mechanisms of robust multistability

Through the above analysis, we have identified the key factors determining multistability in each framework. Next, we aim to intuitively understand why these factors could enhance multistability robustness. To achieve this, we select a representative circuit from the 729 circuits and perform bifurcation analysis to uncover the underlying dynamical mechanism.

**Fig. 6:**
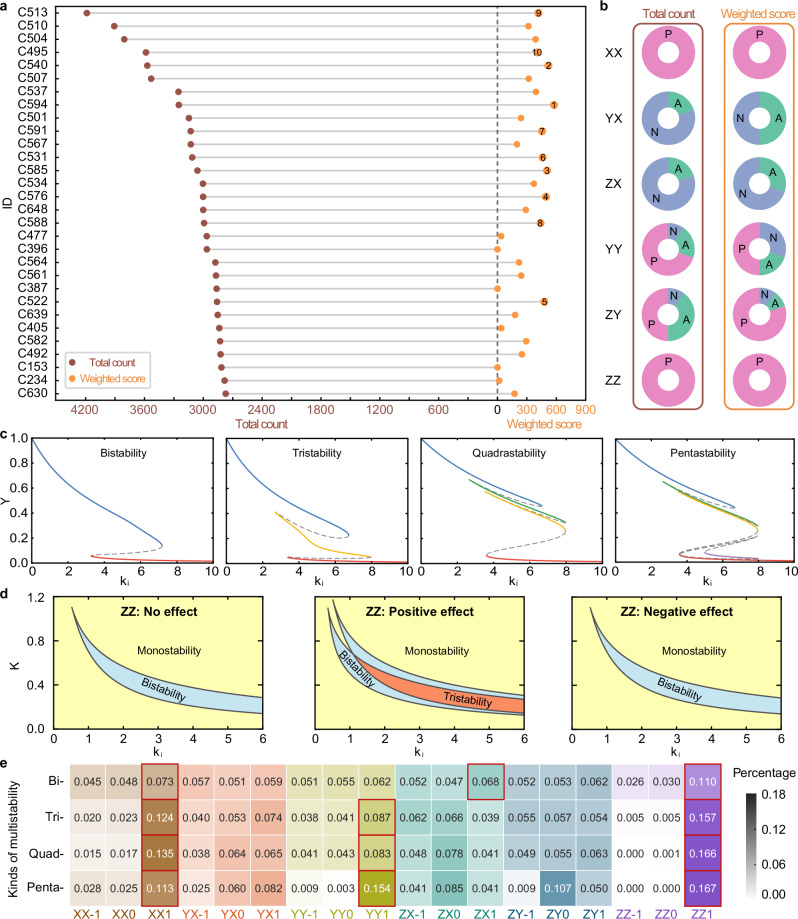
Representative circuit analysis and global statistical evaluation of multistability in the CFL-AND framework. **a** The top 30 circuits ranked by total count (brown dots), defined as the number of parameter sets generating multistability out of 100,000. Weighted score (orange dots) incorporates the contribution of higher-order stable states, calculated as 3 × count of tristability + 4 × count of quadrastability + 5 × count of pentastability. **b** The distribution of regulatory effects—positive (P), negative (N), and absent (A)—for the six feedback edges is shown for the top 10 circuits, ranked by total count (left panel) and weighted score (right panel). **c** One-parameter bifurcation diagrams of the representative circuit (C513) in the CFL-AND framework with respect to k_i_. Using the parameter sets listed in Supplementary Table S2, the system exhibits bistability, tristability, quadrastability, and pentastability, respectively. Solid curves denote stable steady states and dashed curves denote unstable steady states. **d** Two-parameter bifurcation analysis of C513 shown in the k_i_-K parameter space. The analysis is performed based on the tristable parameter set in Supplementary Table S2. The gray curves delineate the boundaries between monostability, bistability, and tristability, which are shaded in yellow, blue, and orange, respectively. From left to right, the three panels illustrate the effects of ZZ regulation: no effect, positive effect, and negative effect. Except for the sign of ZZ regulation, all other parameters remain identical. k_i_, the common inactivation rate constant of all three nodes; K, the common half-saturation constant of all regulatory interactions. **e** Global statistical dependency between feedback regulation and different kinds of multistability in the CFL-AND framework. The horizontal axis represents the 18 regulatory configurations of the six feedback edges (XX, YX, ZX, YY, ZY, and ZZ), each taking activation (1), absence (0), or inhibition (−1). The vertical axis denotes bistability, tristability, quadrastability, and pentastability. For each regulatory configuration, percentages are calculated by counting the parameter sets generating a given stability type and then normalizing within each multistability category (row-wise normalization). Red boxes highlight the top three regulatory configurations with the highest percentages for each stability type.

To select the representative circuit, we first identify the top 30 circuits based on their total count—the number of parameter sets (out of 100,000 predefined sets) that generate multistability, following the robustness evaluation procedure described in the Circuit Enumeration and Multistable Robustness Assessment section. The top 30 circuits ranked by total count and their corresponding weighted scores are shown in Fig. 6a. Next, we calculate the weighted scores, defined as 3 × the count of tristability + 4 × the count of quadrastability + 5 × the count of pentastability, thereby assigning greater weight to circuits with a higher number of stable states. We then analyze the distribution of the three regulatory effects (positive, negative, and absent) across the six feedback edges for the top 10 circuits ranked by both total count and weighted score, as shown in Fig. 6b. To determine the regulatory effect of each feedback edge for the representative circuit, we adopt a majority-rule approach. Notably, in the CFL-AND framework, both ranking methods converge on the same representative circuit—C513—with XX, YY, ZY, and ZZ as positive, and YX and ZX as negative.

For C513, the representative circuit of the CFL-AND framework, we perform both one-parameter and two-parameter bifurcation analyses. In the one-parameter analysis, the common inactivation rate constant of all three nodes (k_i_) is chosen as the bifurcation parameter, while all other parameters remain fixed at the values listed in Supplementary Table S2. By varying k_i_, C513 can exhibit bistability, tristability, quadrastability, and pentastability under different parameter sets (Fig. 6c), and the corresponding parameter combinations are provided in Supplementary Table S2. Based on a parameter set that generates tristability, we further conduct a two-parameter bifurcation analysis under three conditions: ZZ exhibits no effect, a positive effect, or a negative effect. In this analysis, we simultaneously vary k_i_ and the common half-saturation constant of all regulatory interactions (K), while all other parameters are kept identical. As shown in Fig. 6d, when ZZ exerts no effect or a negative effect, the circuit can only achieve bistability in a relatively narrow region of the (k_i_, K) parameter space. In contrast, a positive effect of ZZ substantially enlarges the parameter region supporting multistability and even enables a significant area of tristability. This result demonstrates the critical role of Z self-activation in expanding the multistable region and facilitating higher-order stable states, offering insights into its contribution to multistability robustness.

To further assess how feedback regulation globally influences multistability in the CFL-AND framework, we analyze the multistability outcomes across all 729 circuits, each sampled with 100,000 parameter sets. Figure 6e summarizes the dependency of bistability, tristability, quadrastability, and pentastability on different regulatory configurations of the six feedback edges. For bistability, the highest contributions arise from Z self-activation (ZZ1), X self-activation (XX1), and activation of X by Z (ZX1). For tristability, quadrastability, and pentastability, the dominance of Z self-activation becomes even more pronounced. In addition, X self-activation and Y self-activation (YY1) consistently rank among the most influential configurations. Overall, these results indicate that self-activation—especially Z self-activation, followed by X and Y self-activation—is strongly enriched in circuits capable of generating different kinds of multistability.

Using the same procedure, we identify representative circuits for the other three frameworks: CFL-OR, IFL-AND, and IFL-OR, which are C669, C723, and C507, respectively (Supplementary Fig. S2). For each representative circuit, two-parameter bifurcation analyses are performed as described for C513. In all cases, positive self-feedback at the key feedback edge(s) markedly broadens the parameter region supporting multistability (not shown). In addition, global statistical analyses analogous to Fig. 6e are carried out for these three frameworks. Despite differences in feedforward type (CFL vs IFL) and logical integration (AND vs OR), the enrichment pattern remains consistent: self-activation is preferentially associated with circuits capable of generating different kinds of multistability (not shown). These findings collectively establish positive self-feedback as a general mechanism underlying robust multistability in across all four frameworks.

### Comparative analysis of multistability across four frameworks

**Fig. 7:**
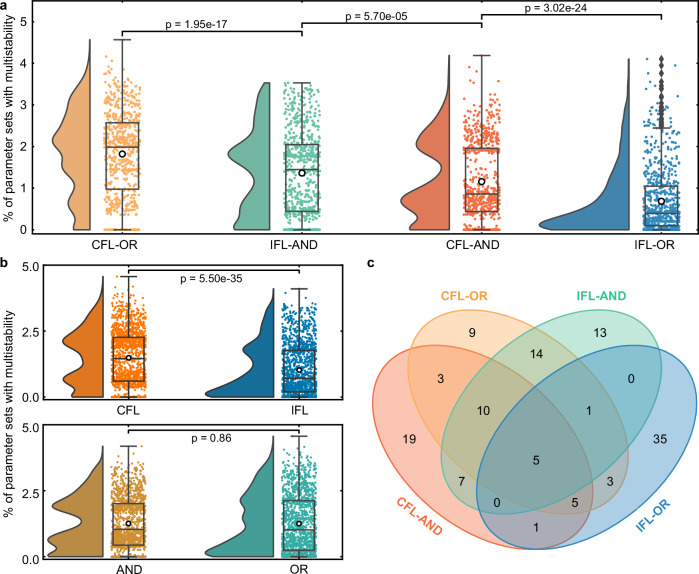
Statistical comparison of multistability prevalence and intersection of top-ranking circuits across four frameworks. **a** Distribution of multistability prevalence across 729 circuits, showing that CFL-OR exhibits the highest prevalence, followed by IFL-AND, CFL-AND, and IFL-OR (p-values indicated in the panel). **b** Comparison of circuit groups: the upper panel shows that coherent (CFL) and incoherent (IFL) feedforward loops differ significantly in their multistability prevalenc (*p* < 0.001), while the lower panel shows that AND- and OR-type logic display no significant difference (*p* = 0.86, Wilcoxon rank-sum test). **c** The Venn diagram illustrating the overlap among the top 50 circuits ranked by *Robustness Percentage* for each of the four frameworks.

This section presents a statistical comparison of the 729 circuits across the four frameworks. In Fig. 7a, the four frameworks are ranked by median multistability prevalence—CFL-OR exhibits the highest prevalence, followed by IFL-AND, CFL-AND, and finally IFL-OR—with statistically significant differences across groups. As shown in Fig. 7b, circuits with coherent feedforward loop (CFL) generally achieve significantly higher multistability than those with incoherent feedforward loop (IFL), whereas the difference between AND-type and OR-type circuits is comparatively modest. Overall, these results underscore that feedforward loop type (CFL vs. IFL) is the dominant factor determining multistability, while choice of logic gate (AND vs. OR) exerts a less pronounced influence on robustness of multistable behavior.

To further investigate the consistency of high-performing designs across the four frameworks, we analyzed the intersection of the top 50 circuits ranked by *Robustness Percentage* for each framework. As shown in the Venn diagram (Fig. 7c), only five circuits are shared across all four frameworks, indicating that despite similar evaluation criteria, the optimal designs differ substantially across circuit structures and logic implementations. These five consistently high-ranking circuits include C504, C507, C510, C534, and C588. Notably, when the threshold is made more stringent to include only the top 30 circuits, C507 emerges as the only one present across all frameworks, highlighting its exceptional robustness under diverse topological and logical conditions. This finding suggests that while overall circuit topology strongly influences multistability, the robustness of certain well-structured circuits can transcend specific feedforward loop types and logic gate configurations.

## Discussion

**Fig. 8:**
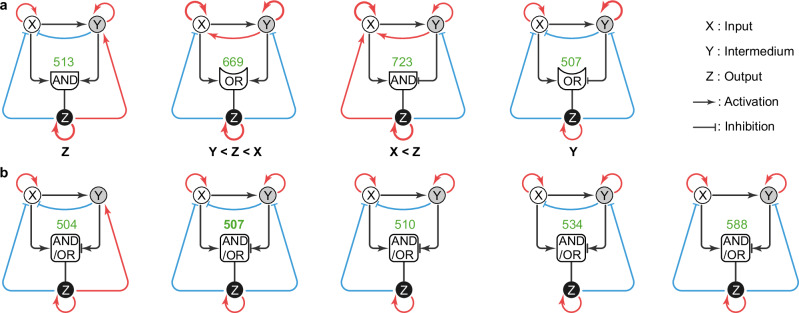
Representative circuits and common high-performing designs across FFL frameworks. **a** Representative circuits for each framework: coherent FFL with AND logic (C513), coherent FFL with OR logic (C669), incoherent FFL with AND logic (C723), and incoherent FFL with OR logic (C507). For each framework, the primary factor promoting multistability is highlighted: self-activation at the output node (CFL-AND); strongest contribution from the input node, followed by the output and intermediate nodes (CFL-OR); combined input and output self-activation, with the latter being more influential (IFL-AND); and self-activation of the intermediate node (IFL-OR). The relative magnitudes of these contributions are presented in Supplementary Fig. S3. **b** Common high-performing designs appearing in the top 50 for all four frameworks: C504, C507, C510, C534, and C588, with C507 uniquely also ranking within the top 30 in every framework. Node labels: X, input; Y, intermediate; Z, output. Arrowheads and blunt ends denote activation and inhibition, respectively.

Through large-scale simulations of 729 circuit topologies across four types of FFL frameworks, we identify two key principles underlying the emergence of multistability. First, while self-activation is a fundamental mechanism promoting multistability, the key contributing nodes vary among FFL frameworks (Figs. 3–5, Supplementary Fig. S3). As summarized in Fig. 8a, coherent FFLs with AND logic rely most heavily on self-activation at the output node, whereas in coherent FFLs with OR logic, all three nodes contribute, with the input node being most influential. In incoherent FFLs, the dominant contributor shifts: for AND logic, both input and output self-activation enhance multistability, with the latter being more influential; for OR logic, self-activation of the intermediate node is most critical. Furthermore, we identify the representative high-performing circuit for each framework: C513, C669, C723, and C507 (Fig. 6, Supplementary Fig. S2). Bifurcation analysis of these designs confirms that self-activation at the critical node expands the parameter space supporting multistability, thereby providing a mechanistic basis for the statistical determinants identified above.

Second, the capacity to generate multistability varies substantially among the four FFL types, with coherent loops generally outperforming incoherent ones, while the choice of logic gate (AND vs. OR) exerts a comparatively weaker effect on the robustness of multistable behavior (Fig. 7). Despite these differences, a small set of circuits consistently ranks among the top performers. As shown in Fig. 8b, five circuits (C504, C507, C510, C534, and C588) consistently appear among the top 50 designs across all frameworks, suggesting that certain designs possess robust multistability regardless of specific topologies or logic types. Among these, C507 is unique in ranking within the top 30 in every framework. These findings indicate that while circuit architecture fundamentally influences multistability, a few versatile designs can transcend structural constraints and serve as generalizable templates for robust multistability across diverse regulatory contexts.

Placed in a broader context, it is useful to consider how these findings relate to earlier systematic explorations of network topology and dynamics. Following the pioneering work of Ma et al., who exhaustively searched all three-node enzymatic network topologies and scanned the parameters of their dynamic equations to identify designs capable of biochemical adaptation^51^, researchers have applied similar approaches to probe other dynamic behaviors. These include switch-like responses^52^, oscillatory behavior^50^, as well as the independent dynamics of adaptation and oscillation in the presence of biological noise^53,54^. For instance, Shah and Sarkar exhaustively explored all possible two- and three-component topologies that produce switch-like cellular responses, assessing their robustness in terms of ultrasensitivity and bistability across enzymatic, transcriptional, and hybrid networks^52^. Likewise, Li et al. generated a comprehensive atlas of two- and three-node networks and systematically analyzed the relationship between network structure and oscillatory robustness^50^. Despite these advances, multistability—another fundamental dynamic behavior—has received comparatively little systematic attention. This gap motivates the present work, which applies large-scale, topology-wide screening to assess and compare the robustness of multistable circuits across diverse FFL frameworks. Our work thus complements these studies by extending the exploration of design principles to multistability.

Another relevant methodological reference for the present study is the RACIPE approach^55^, which also explores circuit dynamics using Hill-type equations combined with randomized kinetic parameters. However, the two methods differ in three important aspects, including the formulation of regulatory terms, the strategy for parameter sampling, and the procedure used to compute and identify steady states (described in Supplementary Note 2). To examine whether our main conclusions are method-dependent, we further test four representative circuits (C513, C669, C723, and C507) using a RACIPE-based validation scheme. When the same RACIPE equation formulation and identical 10,000 sampled parameter sets are used, the multistability probabilities estimated by the RACIPE procedure and by our quasi-Newton–based method are highly consistent (Supplementary Table S3). This consistency supports the reliability of the quasi-Newton–based steady-state identification used in our study. Although the probabilities of multistability may depend on the specific parameter ranges and equation formulations employed, the strong agreement between the two approaches indicates that the main structure–dynamics relationships identified here are robust to these methodological differences.

Despite the breadth of our systematic analysis, several limitations warrant discussion. First, although eight distinct FFL types have been described in theory, we restrict our investigation to the coherent type-1 (C1-FFL) and incoherent type-1 (I1-FFL) architectures, each implemented with AND- and OR-gate logic. This choice reflects both the disproportionately high prevalence of these two FFL types in natural systems and the computational cost associated with exhaustive parameter screening. Second, because auto-regulatory loops are relatively rare in enzymatic networks, we model each node in our circuits as a transcriptional component, thereby focusing on transcriptional regulatory networks and excluding enzymatic implementations. Third, while coupled FFL–FBL structures have been documented in diverse biological contexts, previous studies have not necessarily examined their capacity for multistability, and many of the coupled loops capable of producing multistability may not conform to the two FFL types considered here. As a result, it remains challenging to identify natural regulatory networks resembling the high-performing circuit architectures identified here. Consequently, the primary value of the present work lies less in recapitulating naturally occurring circuits and more in providing a systematic design framework for synthetic biology applications.

Notwithstanding these limitations, our results offer concrete and generalizable guidelines for constructing synthetic circuits with robust multistability. Specifically, when the FFL type and logic gate of a target design are known, the representative high-performing circuits identified for each framework—C513 (CFL-AND), C669 (CFL-OR), C723 (IFL-AND), and C507 (IFL-OR)—provide optimal starting points for implementation. Conversely, in situations where such architectural details are unknown or flexible, a small set of top-performing designs—C504, C507, C510, C534, and C588—consistently achieve high multistability across all frameworks. Among these, C507 is particularly notable for its superior performance in every context tested. Overall, this study not only deepens our theoretical understanding of network motifs but also provides a practical blueprint for rationally designing multistable regulatory circuits, with potential utility in synthetic decision-making modules, cellular memory systems, and programmable state transitions.

## Methods

This study focuses on four distinct frameworks: CFL-AND, CFL-OR, IFL-AND, and IFL-OR. In these frameworks, the input node (X) activates both the intermediate node (Y) and the output node (Z). In CFL frameworks, Y activates Z, whereas in IFL frameworks, Y inhibits Z. With these three connections specified, the circuits are further defined by six additional directed edges among the three nodes. Each edge can represent positive, negative, or absent regulation, resulting in 3^6^ = 729 possible circuits for every framework.

We treat each node in the circuit as a transcription factor. In biological systems, transcription factors frequently switch between transcriptionally active and inactive states through processes such as phosphorylation, conformational changes, ligand binding, cofactor association, or nuclear localization. Because these processes typically occur on a faster timescale than protein synthesis and degradation, the total concentration of each transcription factor can be approximated as constant over the timescale of the regulatory dynamics. Accordingly, we normalize the total concentration to unity. The variable ‘A’ represents the fraction of the transcription factor in the active state, while ‘1 - A’ represents the inactive fraction. In our model, the regulatory dynamics of each transcription factor are governed by three types of transitions: basal activation-inactivation, activator-mediated activation, and inhibitor-mediated inactivation^50,56^. Based on these processes, the temporal evolution of the active fraction is described by the following dynamical equations:

1 $$\begin{array}{ll}\frac{{{(1-X)Y{^n}}}}{{(K^n + Y^n)}} & ={k}_{a}(1-X)-{k}_{i}X\\ & +\,{k}_{XX}\frac{{\sigma }_{11}({\sigma }_{11}+1)}{2}(1-X)\frac{{X}^{n}}{{K}^{n}+{X}^{n}}-{k}_{XX}\frac{{\sigma }_{11}({\sigma }_{11}-1)}{2}X\frac{{X}^{n}}{{K}^{n}+{X}^{n}}\\ & +\,{k}_{YX}\frac{{\sigma }_{21}({\sigma }_{21}+1)}{2}{(1-X)}^{n}-{k}_{YX}\frac{{\sigma }_{21}({\sigma }_{21}-1)}{2}X\frac{{Y}^{n}}{{K}^{n}+{Y}^{n}}\\ & +\,{k}_{ZX}\frac{{\sigma }_{31}({\sigma }_{31}+1)}{2}(1-X)\frac{{Z}^{n}}{{K}^{n}+{Z}^{n}}-{k}_{ZX}\frac{{\sigma }_{31}({\sigma }_{31}-1)}{2}X\frac{{Z}^{n}}{{K}^{n}+{Z}^{n}}\end{array}$$

2 $$\begin{array}{ll}\frac{{\rm{d}}Y}{{\rm{d}}t} & ={k}_{a}(1-Y)-{k}_{i}Y\\ & +\,{{\rm{k}}}_{XY}(1-Y)\frac{{X}^{n}}{{K}^{n}+{X}^{n}}\\ & +\,{k}_{YY}\frac{{\sigma }_{22}({\sigma }_{22}+1)}{2}(1-Y)\frac{{Y}^{n}}{{K}^{n}+{Y}^{n}}-{k}_{YY}\frac{{\sigma }_{22}({\sigma }_{22}-1)}{2}Y\frac{{Y}^{n}}{{K}^{n}+{Y}^{n}}\\ & +\,{k}_{ZY}\frac{{\sigma }_{32}({\sigma }_{32}+1)}{2}(1-Y)\frac{{Z}^{n}}{{K}^{n}+{Z}^{n}}-{{\rm{k}}}_{ZY}\frac{{\sigma }_{32}({\sigma }_{32}-1)}{2}Y\frac{{Z}^{n}}{{K}^{n}+{Z}^{n}}\end{array}$$

3 $$\begin{array}{ll}\frac{{\rm{d}}Z}{{\rm{d}}t} & ={k}_{a}(1-Z)-{k}_{i}Z\\ & +\,{k}_{XYZ}(1-Z)T\\ & +\,{k}_{ZZ}\frac{{\sigma }_{33}({\sigma }_{33}+1)}{2}(1-Z)\frac{{Z}^{n}}{{K}^{n}+{Z}^{n}}-{k}_{ZZ}\frac{{\sigma }_{33}({\sigma }_{33}-1)}{2}Z\frac{{Z}^{n}}{{K}^{n}+{Z}^{n}}.\end{array}$$

Here, k_a_ and k_i_ denote the basal activation and inactivation rate constants, respectively. σ_ji_ defines the regulatory effect of node j on node i, where i, j = 1, 2, 3 (1 for X, 2 for Y, and 3 for Z). It takes values –1, 0, or 1, indicating inhibition, no regulation, and activation, respectively. The parameter k_ij_ is the maximal regulatory rate, K is the half-saturation constant, and n is the Hill coefficient. The detailed derivation of these dynamical equations under the activation-inactivation framework is provided in the Supplementary Note 3.

The term T in Eq. (3), which captures the combined regulatory influence of X and Y on Z, varies across the four distinct frameworks. Detailed expressions and derivations are provided in Supplementary Note 4. In the CFL-AND case,

$$T=\frac{{X}^{n}}{{K}^{n}+{X}^{n}}\frac{{Y}^{n}}{{K}^{n}+{Y}^{n}}.$$

In the CFL-OR case,

$$T=1-\frac{{K}^{n}}{{K}^{n}+{X}^{n}}\frac{{K}^{n}}{{K}^{n}+{Y}^{n}}.$$

In the IFL-AND case,

$$T=\frac{{X}^{n}}{{K}^{n}+{X}^{n}}\frac{{K}^{n}}{{K}^{n}+{Y}^{n}}.$$

In the IFL-OR case,

$$T=1-\frac{{K}^{n}}{{K}^{n}+{X}^{n}}\frac{{Y}^{n}}{{K}^{n}+{Y}^{n}}.$$

With the circuit and its governing ODEs established, we proceed to define the parameter values. A total of 100,000 parameter sets are sampled using the Latin hypercube sampling method on a logarithmic scale to efficiently explore the multidimensional parameter space (described in Supplementary Note 5). The model variables represent normalized activity levels ranging between 0 and 1. Accordingly, the half-saturation constant K, which characterizes the activation threshold, is sampled within the range of 0.1–1. The basal activation rate k_a_ is also restricted to a relatively small range of 0.1–1 to represent weak background activation in the absence of regulatory inputs. All other parameters correspond to maximal regulatory rates and are sampled within a broader range of 0.1–10 to account for variability in regulatory strengths across different cellular environments. Notably, the Hill coefficient n is fixed at 4 to facilitate robust and reliable outcomes.

## Supplementary information

Supplementary Information.

Supplementary Data.

## Data Availability

All data generated in this study are available in the supplementary materials.
