# Supplementary material for: Design principles for robust multistability in coupled feedforward-feedback regulatory circuits
Source: NPJ Syst Biol Appl. 2026 May 2;12:102. doi: 10.1038/s41540-026-00731-1 (PMC13341765; doi:10.1038/s41540-026-00731-1)
Supplement: Supplementary file 1 — Supplementary Information. [file 41540_2026_731_MOESM1_ESM.pdf]

Supplementary Information

Fig. S1 Canonical feedforward and feedback loop motifs

Fig. S2 Selection of representative circuits in CFL-OR, IFL-AND, and IFL-OR frameworks

Fig. S3 Individual and pairwise contributions of feedback edges to multistability across four frameworks

Table S1 Statistical association between feedback edges and multistability robustness across four frameworks

Table S2 Parameters used in the study and representative values for circuit C513 in the CFL-AND framework

Table S3 Comparison of multistability probabilities obtained using the RACIPE approach and the quasi-Newton

Note 1 Quasi-Newton Algorithm for Equilibrium Computation

Note 2 Methodological Comparison Between the Present Method and the RACIPE Approach

Note 3 Derivation of the Governing Equation

Note 4 Regulatory Dynamics of Feedforward Loops

Note 5 Latin Hypercube Sampling

Supplementary Data. Robustness percentages, classifications, and connectivity patterns of 729 circuits across four frameworks.

(XLSX)

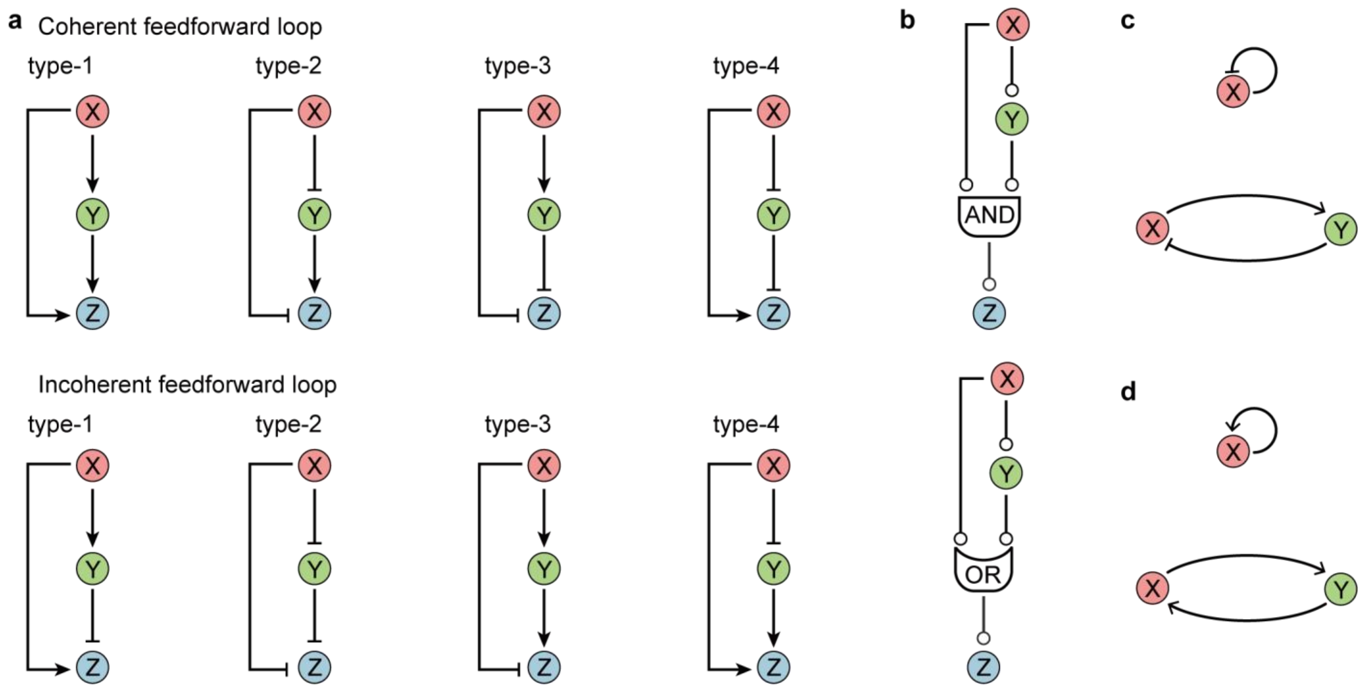

Supplementary Fig. S1. Canonical feedforward and feedback loop motifs. a The eight possible three-node feedforward loops (FFLs), classified into four coherent and four incoherent types, depending on whether the net effect of the indirect path (X→Y→Z) is consistent with or opposite to that of the direct path (X→Z). **b** Two representative input-integration logics at the output of FFLs, AND and OR gates, determine how signals from X and Y are integrated. (**c**) Negative feedback loops and (**d**) positive feedback loops, which can arise either through direct autoregulation or indirectly via intermediate components.

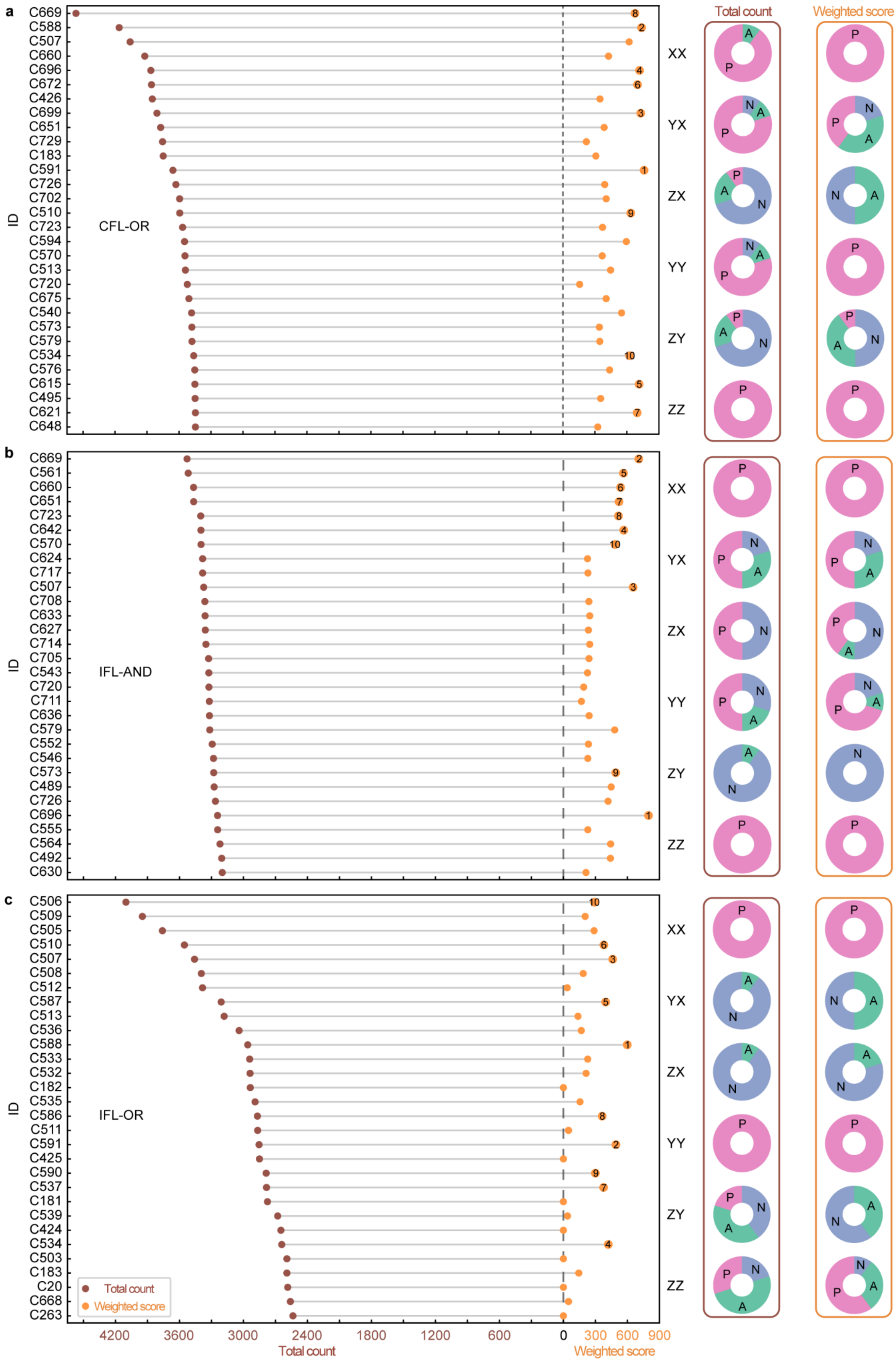

Supplementary Fig. S2. Selection of representative circuits in CFL-OR, IFL-AND, and IFL-OR frameworks. a–c The

top 30 circuits in each framework are ranked by total count (brown dots), defined as the number of parameter sets (out of 100,000) that generate multistability. The weighted score (orange dots) emphasizes circuits with higher-order multistability and is calculated as 3 × count of tristability + 4 × count of quadrastability + 5 × count of pentastability. The distribution of regulatory effects—positive (P), negative (N), and absent (A)—for the six feedback edges is shown for the top 10 circuits, ranked by total count (left panel) and by weighted score (right panel). Representative circuits for the CFL-OR, IFL-AND, and IFL-OR frameworks are identified as C669, C723, and C507, respectively.

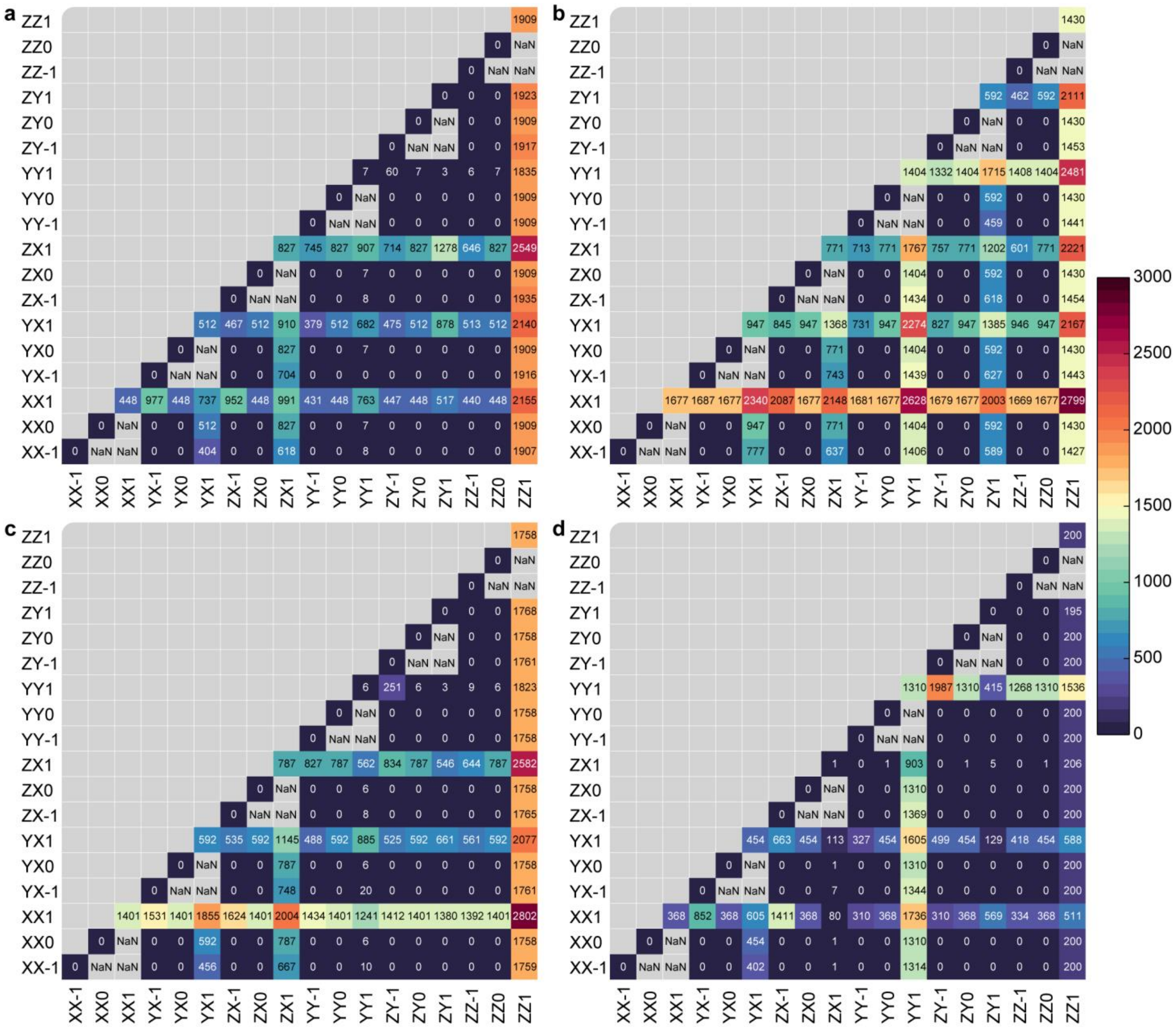

Supplementary Fig. S3. Individual and pairwise contributions of feedback edges to multistability across four frameworks. Heatmaps show the number of parameter sets (out of 100,000) that give rise to multistability for different regulatory effects of six feedback edges: XX (X on itself), YX (Y on X), ZX (Z on X), YY (Y on itself), ZY (Z on Y), and ZZ (Z on itself). Each edge can take one of three regulatory effects: activation (1), absence (0), or inhibition (−1). Diagonal entries indicate the impact of individual feedback edges, while off-diagonal entries capture pairwise effects. Larger values correspond to stronger multistable robustness. Across frameworks, distinct edges play dominant roles: in CFL-AND (**a**), self-activation of Z exerts the strongest single-edge effect; in CFL-OR (**b**), self-activation of X contributes most, followed by Z and then Y; in IFL-AND (**c**), both X and Z self-activation enhance multistability, with Z being more influential; and in IFL-OR (**d**), self-activation of Y dominates.

Table S1. Statistical association between feedback edges and multistability robustness across four frameworks

Type	Edge	χ^2	Significance	Cramer's *V*
CFL-AND	**ZZ**	**211.9251**	***** (*p*<0.001)**	**0.9793**
	**ZX**	**119.7016**	***** (*p*<0.001)**	**0.736**
	**XX**	**111.4445**	***** (*p*<0.001)**	**0.7101**
	ZY	8.5625	* (*p*<0.05)	0.1968
	YX	5.5075	ns (*p*≥0.05)	0.1579
	YY	5.1145	ns (*p*≥0.05)	0.1521
CFL-OR	**ZZ**	**111.4615**	***** (*p*<0.001)**	**0.7338**
	**XX**	**99.4357**	***** (*p*<0.001)**	**0.6931**
	**YY**	**71.2761**	***** (*p*<0.001)**	**0.5868**
	YX	43.3718	*** (*p*<0.001)	0.4577
	ZY	33.5441	*** (*p*<0.001)	0.4026
	ZX	29.715	*** (*p*<0.001)	0.3789
IFL-AND	**ZZ**	**251**	***** (*p*<0.001)**	**1**
	**XX**	**208.0745**	***** (*p*<0.001)**	**0.9105**
	YX	47.405	*** (*p*<0.001)	0.4346
	ZX	37.5077	*** (*p*<0.001)	0.3866
	ZY	2.0731	ns (*p*≥0.05)	0.0909
	YY	0.6442	ns (*p*≥0.05)	0.0507
IFL-OR	**YY**	**373**	***** (*p*<0.001)**	**1**
	XX	56.4761	*** (*p*<0.001)	0.3891
	ZX	30.5475	*** (*p*<0.001)	0.2862
	YX	7.6773	* (*p*<0.05)	0.1435
	ZY	4.0238	ns (*p*≥0.05)	0.1039
	ZZ	0.8098	ns (*p*≥0.05)	0.0466

Note: The χ^2 test evaluates whether regulatory effect (positive / none / negative) and robustness category (High vs. Low) are statistically independent. Significance levels are denoted as *** (p < 0.001), * (p < 0.05), and ns ($p \geq 0.05$). Because statistical significance does not quantify the strength of association, Cramer's V is used to measure the magnitude of association between edge regulation and robustness category, with values closer to 1 indicating stronger association. Edges highlighted in **bold** correspond to the largest Cramer's *V* within each framework and are therefore interpreted as the primary associated factors distinguishing high- and low-robustness circuit groups.

Table S2. Parameters used in the study and representative values for circuit C513 in the CFL-AND framework

Parameter	Description	Bistability	Tristability	Quadrastability	Pentastability
k_a	Basal activation rate constant	0.15			
k_i	Basal inactivation rate constant	5			
k_{XX}	The maximal regulatory rate of X on itself	4.5			
k_{YX}	The maximal regulatory rate of Y on X	0.15			
k_{ZX}	The maximal regulatory rate of Z on X	0.5			
k_{XY}	The maximal regulatory rate of X on Y	1.5			
k_{YY}	The maximal regulatory rate of Y on itself	1.5		5	5
k_{ZY}	The maximal regulatory rate of Z on Y	0.5			0.14
k_{ZZ}	The maximal regulatory rate of Z on itself	1.5	5	5	5
k_{XYZ}	The maximal joint regulatory rate of X and Y on Z	1.5			
n	Hill coefficient	3			
K	Half-saturation constant	0.25			

Note: All parameters have the same meanings across the four frameworks (CFL-AND, CFL-OR, IFL-AND, and IFL-OR). The parameter values listed here correspond to the representative circuit C513 in the CFL-AND framework. For clarity and to avoid redundancy, the complete parameter set is listed only for the bistable case. The columns corresponding to tristability, quadrastability, and pentastability display only the parameters whose values differ from those used in the bistable case; parameters left blank in these columns take the identical values as in the bistable case. For the one-parameter bifurcation diagrams in Fig. 6c, the parameter sets corresponding to bistability, tristability, quadrastability, and pentastability are used, respectively, while varying k_i. For the two-parameter bifurcation diagrams in Fig. 6d, the analysis is performed by varying k_i and K based on the tristable parameter set in this table.

Table S3. Comparison of multistability probabilities obtained using the RACIPE approach and the quasi-Newton method for representative circuits

	C513	C669	C723	C507
P_R	32.68%	35.47%	58.14%	62.43%
P_N	32.87%	36.43%	57.18%	62.97%

Note: P_R and P_N denote the probabilities of producing multistability estimated using the RACIPE approach and the quasi-Newton–based method, respectively.

Quasi-Newton Algorithm for Equilibrium Computation

Consider an n-dimensional nonlinear system

$$f(X)=0,$$

where $X \in \mathbb{R}^n$ denotes the vector of unknown variables, and $f:\mathbb{R}^n \to \mathbb{R}^n$ is a nonlinear vector-valued function. To numerically solve the system, a first-order Taylor expansion of $f(X)$ is performed about an initial iterate $X^{(0)}$, yielding the linear approximation

$$f(X) \approx f(X^{(0)}) + J(X^{(0)})(X - X^{(0)}),$$

where $J(X^{(0)})$ denotes the Jacobian matrix evaluated at $X^{(0)}$. Solving the resulting linearized system leads to

$$X = X^{(0)} - J^{-1}(X^{(0)}) f(X^{(0)}).$$

This formulation is extended to an iterative scheme as

$$X^{(k+1)} = X^{(k)} - J^{-1}(X^{(k)}) f(X^{(k)}),$$

where $X^{(k)}$ denotes the variable vector at the k-th iteration, $J(X^{(k)})$ is the corresponding Jacobian matrix, and $f(X^{(k)})$ represents the function value vector at this iteration.

In the quasi-Newton method, explicit evaluation of partial derivatives in the Jacobian matrix is avoided. Instead, the Jacobian matrix is approximated by a divided-difference matrix constructed from finite variations in function values. This divided-difference matrix, denoted by $F(X^{(k)})$, serves as a numerical approximation of the Jacobian matrix $J(X^{(k)})$.

To avoid the computational cost associated with explicit matrix inversion, the correction vector $\delta^{(k)}$ is defined as $\delta^{(k)} = F^{-1}(X^{(k)}) f(X^{(k)})$. This leads to the linear system

$$f(X^{(k)}) = F(X^{(k)})\delta^{(k)}.$$

At the k-th iteration, both the function value vector $f(X^{(k)})$ and the divided-difference matrix $F(X^{(k)})$ are known, while the correction vector $\delta^{(k)}$ is the only unknown. The original problem is thus reduced to solving a system of linear equations. The iteration is then completed using the update formula

$$X^{(k+1)} = X^{(k)} - \delta^{(k)}.$$

Convergence is assessed using the Euclidean norm of the function value vector, with the convergence

criterion defined as

$$\left\| f(X^{(k)}) \right\|_2 < \varepsilon,$$

where ε is a prescribed tolerance threshold. When this condition is satisfied, the current iterate is considered sufficiently close to the true solution and the iteration is terminated. Otherwise, the update procedure is repeated until convergence is achieved.

To obtain all possible numerical solutions, the algorithm is initialized from multiple starting points throughout the variable space. Candidate solutions converging from different initial values are filtered using a Euclidean-norm-based criterion with a prescribed tolerance threshold, resulting in a set of unique solutions.

Methodological Comparison Between the Present Method and the RACIPE Approach

Another relevant modeling approach for exploring the dynamical behaviors of gene regulatory networks is the Random Circuit Perturbation (RACIPE) approach. RACIPE employs Hill-type regulatory equations and randomizes kinetic parameters over biologically plausible ranges to generate an ensemble of models, whose steady states are then analyzed to characterize the dynamical repertoire of a given network topology. In this respect, our approach shares certain conceptual similarities with RACIPE, particularly in the use of parameter randomization and nonlinear regulatory functions to probe multistable behavior. However, important methodological differences exist in three key aspects: (i) the construction of regulatory terms in the dynamical equations, (ii) the parameter sampling strategy, and (iii) the computation and identification of steady states. Below we compare the two approaches.

Equation formulation

In the RACIPE approach, regulatory interactions are represented using nonlinear shifted Hill functions, and the effects of multiple regulatory inputs are integrated multiplicatively. Specifically, the production rate of a target node is written as the product of a maximal production rate and the shifted Hill functions corresponding to all incoming regulatory edges. Each shifted Hill function contains parameters such as the Hill coefficient, the half-saturation constant, and a fold-change parameter that determines the maximal regulatory strength. A fold change larger than one represents activation, whereas a fold change smaller than one represents inhibition.

The modeling approach used in the present study also adopts Hill-type nonlinear regulatory functions but organizes the regulatory terms differently in the dynamical equations. The regulatory effects are represented through additive combinations of Hill-type terms. Furthermore, because the present study considers both AND-type and OR-type feedforward loops, two corresponding forms of regulatory equations are constructed to distinguish these logical structures. Specifically, for AND-type regulation, the effective regulatory term is represented as the product of the corresponding Hill activation functions, whereas for OR-type regulation the interaction is formulated as the complement of the product of inhibitory Hill functions.

Parameter sampling strategy

The primary objective of RACIPE is to identify gene expression patterns that are robust to parameter variations for a fixed network topology. To achieve this goal, RACIPE introduces the half-functional rule, which constrains threshold parameters such that each regulatory edge has an approximately 50% probability of being functional across the ensemble of randomly generated models. After determining these threshold ranges, other

parameters are randomly sampled within predefined intervals to generate a large ensemble of models. This strategy statistically enhances the connection between network topology and robust dynamical patterns.

In contrast, the present study employs Latin hypercube sampling to explore the parameter space without enforcing additional constraints such as the half-functional rule. Compared with simple random sampling, Latin hypercube sampling provides a stratified sampling scheme that ensures a more uniform coverage of each parameter dimension. This approach divides the range of each parameter into equally probable intervals and guarantees that each interval is sampled exactly once across the ensemble. As a result, the sampled parameter sets achieve a more balanced and space-filling representation of the multidimensional parameter space. This sampling design reduces redundancy in parameter exploration and facilitates systematic comparisons of dynamical behaviors across different circuit topologies.

Steady-state computation and identification

The most important difference between the two approaches lies in the computation and identification of steady states. In RACIPE, the computation of stable states for each parameter set is performed by numerical integration from multiple random initial conditions. After the trajectories converge within a finite simulation time, the terminal states are grouped using a distance-based clustering criterion. Terminal states that are sufficiently close in phase space are treated as the same steady state, and the number of clusters is interpreted as the number of stable steady states.

In contrast, the approach adopted in this study directly computes equilibrium points by solving the steady-state equations using a quasi-Newton root-finding algorithm with multiple initial guesses. The solutions obtained from different initial conditions are then filtered to remove duplicate equilibria. Once the equilibrium points are identified, the stability of each equilibrium is determined by evaluating the eigenvalues of the Jacobian matrix. The number of stable steady states is defined as the number of equilibria whose eigenvalues all have negative real parts. This procedure provides a direct dynamical characterization of fixed-point stability and avoids potential ambiguities associated with trajectory-based clustering approaches.

RACIPE-based method validation

The two approaches emphasize different aspects of the structure–dynamics relationship. RACIPE typically focuses on the range of dynamical behaviors that can arise from a given circuit topology, whereas our study performs exhaustive enumeration of circuit architectures based on feedforward loops combined with feedback regulation and systematically compares their multistability robustness across a large topology space. Because

the two approaches address different research objectives, it is not practical to evaluate all enumerated circuit structures using the RACIPE approach. Therefore, we select four representative circuit topologies (C513, C669, C723, and C507) and perform robustness tests using the RACIPE approach. To ensure a fair comparison, we adopt the equation formulation used in RACIPE. Under this setting, the comparison focuses primarily on differences in the procedures used to compute and identify steady states.

Specifically, for a given circuit, the RACIPE approach first constructs the corresponding dynamical equations according to its modeling rules and generates a set of 10,000 parameter samples. Among these parameter sets, RACIPE identifies those that produce multistability (e.g., bistability, tristability, and higher-order multistability), from which the probability of generating multistability can be estimated (denoted as P_R). To enable a direct comparison, we use the same equation formulation as in RACIPE together with the identical set of 10,000 parameter samples, and apply the quasi-Newton–based procedure for steady-state computation and identification used in our study. Using this procedure, we similarly identify the parameter sets that generate multistability and estimate the corresponding probability of generating multistability (denoted as P_N).

The multistability probabilities obtained by the two approaches are then statistically compared. The results, summarized in Table S3, show that the probabilities of multistability computed by the two approaches are nearly identical when the same equations and parameter sets are used. This agreement demonstrates the reliability of the quasi-Newton–based method used in our study for detecting multistable behavior.

Derivation of the Governing Equation

1. Basal activation-inactivation dynamics

Neglecting synthesis and degradation, we focus on the reversible basal activation-inactivation dynamics of node X. The reaction scheme is given by

$$X_{active} \underset{k_a}{\overset{k_i}{\rightleftarrows}} X_{inactive}.$$

According to the law of mass action, the corresponding dynamical equation is

$$\left.\frac{d[X_{active}]}{dt}\right|_{basal} = k_a[X_{inactive}] - k_i[X_{active}]. \qquad (1)$$

2. Activation dynamics

If protein Y activates X by binding to its inactive form, the reaction can be written as

$$X_{inactive} + nY \underset{k_{-1}}{\overset{k_1}{\rightleftarrows}} X_{bind}Y_n \xrightarrow{k_2} X_{active} + nY.$$

When binding and unbinding are fast compared with the timescale of X activation, the rapid equilibrium approximation yields

$$\frac{d[X_{bind}Y_n]}{dt} = k_1[X_{inactive}][Y]^n - (k_{-1}+k_2)[X_{bind}Y_n] = 0.$$

Under this approximation, the dynamical equation reduces to

$$[X_{inactive}] = \frac{k_{-1}+k_2}{k_1}\frac{[X_{bind}Y_n]}{[Y]^n}.$$

In the absence of synthesis and degradation, mass conservation implies that

$$[X_{bind}Y_n] = [X_{total}] - [X_{active}] - [X_{inactive}].$$

Substitution of the expression for $[X_{inactive}]$ into the above equation followed by rearrangement yields

$$[X_{bind}Y_n] = ([X_{total}] - [X_{active}])\frac{[Y]^n}{K_Y + [Y]^n},$$

where $K_Y = \frac{k_{-1}+k_2}{k_1}$ is the dissociation constant. The activation of X by protein Y can therefore be expressed as

$$\left.\frac{d[X_{active}]}{dt}\right|_{activation} = k_2[X_{bind}Y_n] = k_2([X_{total}] - [X_{active}])\frac{[Y]^n}{K_Y + [Y]^n}. \qquad (2)$$

3. Inactivation dynamics

If protein Z inhibits X by binding to its active form, the reaction is written as

$$X_{active} + nZ \underset{k_{-3}}{\overset{k_3}{\rightleftarrows}} X_{bind} Z_n \xrightarrow{k_4} X_{inactive} + nZ \,.$$

Similarly, under the rapid equilibrium approximation, we obtain

$$\frac{d[X_{bind}Z_n]}{dt} = k_3[X_{active}][Z]^n - (k_{-3} + k_4)[X_{bind}Z_n] = 0,$$

which leads to

$$[X_{active}] = \frac{k_{-3} + k_4}{k_3}\frac{[X_{bind}Z_n]}{[Z]^n}.$$

By mass conservation, we have

$$[X_{bind}Z_n] = [X_{total}] - [X_{active}] - [X_{inactive}].$$

Substituting the expression for $[X_{active}]$ and rearranging yields

$$[X_{bind}Z_n] = ([X_{total}] - [X_{inactive}])\frac{[Z]^n}{K_Z + [Z]^n},$$

where $K_Z = \frac{k_{-3} + k_4}{k_3}$ is the corresponding dissociation constant. The inhibition of X by protein Z can thus be written as

$$\left.\frac{d[X_{inactive}]}{dt}\right|_{inhibition} = k_4[X_{bind}Z_n] = k_4([X_{total}] - [X_{inactive}])\frac{[Z]^n}{K_Z + [Z]^n}.$$

Since we focus on the active form, the above function can be rewritten into

$$\left.\frac{d[X_{total}] - [X_{active}]}{dt}\right|_{inhibition} = k_4[X_{active}]\frac{[Z]^n}{K_Z + [Z]^n}.$$

As $[X_{total}]$ is a constant, the equation can be further simplified to

$$\left.\frac{d[X_{active}]}{dt}\right|_{inhibition} = -k_4[X_{active}]\frac{[Z]^n}{K_Z + [Z]^n}. \qquad (3)$$

4. Final governing equation

To facilitate numerical computation and place variables on a unified scale, we normalize the total concentration to 1. Under this normalization, Eqs. (1)-(3) can be written as

$$\left.\frac{dX}{dt}\right|_{basal} = k_a(1 - X) - k_i X \,,$$

$$\left.\frac{dX}{dt}\right|_{activation} = k_2(1 - X)\frac{Y^n}{K_Y + Y^n},$$

$$\left.\frac{dX}{dt}\right|_{inhibition} = -k_4 X \frac{Z^n}{K_Z + Z^n},$$

where X, Y, and Z denote the normalized concentrations of their respective active forms.

Combining basal activation-inactivation with Y-mediated activation and Z-mediated inactivation, the overall governing equation for X is expressed as

$$\frac{dX}{dt} = k_a (1 - X) - k_i X + k_2 (1 - X) \frac{Y^n}{K_Y + Y^n} - k_4 X \frac{Z^n}{K_Z + Z^n}.$$

Assuming identical dissociation constants, this equation can be rewritten as

$$\frac{dX}{dt} = k_a (1 - X) + k_{YX} (1 - X) \frac{Y^n}{K^n + Y^n} - k_{ZX} X \frac{Z^n}{K^n + Z^n} - k_i X.$$

Here, k_{YX} is the maximal rate of Y-mediated X activation, k_{ZX} is the maximal rate of Z-mediated X inactivation, and K is the shared half-saturation constant.

Regulatory Dynamics of Feedforward Loops

This appendix details the mechanisms by which different types of feedforward loops regulate the activation of protein Z (Z^*) and defines the term T, which captures the combined regulatory influence of upstream proteins X (X^*) and Y (Y^*) on Z. For simplicity in the equations, we will use X, Y, and Z to represent the concentrations of their active forms, X^*, Y^*, and Z^*, respectively.

The probabilities for the four possible binding states of Z are given by:

- $\mathbb{P}(Z) = \frac{K_1^{h_1}}{K_1^{h_1}+X^{h_1}} \frac{K_2^{h_2}}{K_2^{h_2}+Y^{h_2}}$ (Z is free)
- $\mathbb{P}(ZX^*) = \frac{X^{h_1}}{K_1^{h_1}+X^{h_1}} \frac{K_2^{h_2}}{K_2^{h_2}+Y^{h_2}}$ (Z is bound by X^*)
- $\mathbb{P}(ZY^*) = \frac{K_1^{h_1}}{K_1^{h_1}+X^{h_1}} \frac{Y^{h_2}}{K_2^{h_2}+Y^{h_2}}$ (Z is bound by Y^*)
- $\mathbb{P}(ZX^*Y^*) = \frac{X^{h_1}}{K_1^{h_1}+X^{h_1}} \frac{Y^{h_2}}{K_2^{h_2}+Y^{h_2}}$ (Z is bound by both X^* and Y^*)

where K_i denotes the half-saturation constant and h_j the Hill coefficient for each binding site. The term **T** represents the effective probability that Z is in a state that can be converted to its active form, Z^*, under specific regulatory logic. According to the mechanism illustrated in the Figure below, we can obtain the activation status of Gene Z under various binding configurations of X^* and Y^* for each feedforward loop type. A checkmark (✓) indicates activation, while an 'X' (×) indicates no activation.

Configuration	Coherent feedforward loop		Incoherent feedforward loop	
	AND	OR	AND	OR
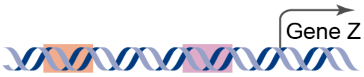	×	×	×	✓
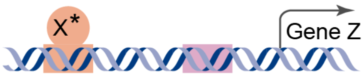	×	✓	✓	✓
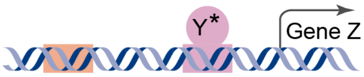	×	✓	×	×
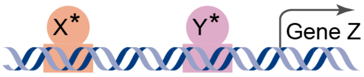	✓	✓	×	✓

1. Coherent Feedforward Loops (CFL)

a. CFL-AND Gate

- **Mechanism**: In this configuration, Gene Z is activated only when both X^* and Y^* are simultaneously bound to its regulatory regions. This corresponds to the ZX^*Y^* binding state. The combined regulatory influence T reflects the probability of Z being in the ZX^*Y^* state.
- **Term T**:
  $T = \frac{X^n}{K^n+X^n} \frac{Y^n}{K^n+Y^n}$

b. CFL-OR Gate

- **Mechanism**: For the OR-gate, Gene Z is activated if at least one of X^* or Y^* (or both) are bound to its regulatory regions. This means any state except the free state Z can lead to activation. The term T represents the probability of Z being in any state other than the free state.

- **Term T**:
  $T = 1 - \frac{K^n}{K^n + X^n} \frac{K^n}{K^n + Y^n}$

---

2. Incoherent Feedforward Loops (IFL)

a. IFL-AND Gate

- **Mechanism**: In this incoherent AND-gate, Gene Z is activated only when X^* is bound to Z, and Y^* is *not* bound. This reflects a scenario where X^* acts as an activator, but Y^* inhibits activation if it is bound. The term T corresponds to the probability of Z being in the ZX^* state, assuming Y^* has an inhibitory effect when bound.
- **Term T**:
  $T = \frac{X^n}{K^n + X^n} \frac{K^n}{K^n + Y^n}$

b. IFL-OR Gate

- **Mechanism**: For the incoherent OR-gate, Gene Z is activated by default or by X^*, but specifically inhibited by Y^*. Thus, all states except for when only Y^* is bound to Z (ZY^*) can lead to activation. The term T represents the probability of Z being in any state except the ZY^* state.
- **Term T**:
  $T = 1 - \frac{K^n}{K^n + X^n} \frac{Y^n}{K^n + Y^n}$

Latin Hypercube Sampling

To efficiently explore the high-dimensional parameter space, parameter sets are generated using Latin hypercube sampling. In this approach, the range of each parameter is divided into N equally probable intervals, and one value is randomly selected from each interval. For parameters spanning multiple orders of magnitude, the sampling occurs in logarithmic space to ensure uniform coverage across orders of magnitude and avoid under-sampling of small values. The sampled values for different parameters are then randomly combined to construct N parameter sets. This ensures that the projection of the N samples onto any dimension is uniformly distributed, requiring far fewer samples than simple random sampling methods.

The Latin hypercube sampling procedure is implemented using the lhs function from the Python package pyDOE, which generates samples uniformly distributed in the interval [0, 1]. To obtain parameters spanning multiple orders of magnitude, a logarithmic mapping is applied to the sampled values. Specifically, a sampled value $a \in [0, 1]$ is first mapped to $b=2(a-0.5)$, yielding $b \in [-1, 1]$. The final parameter value is then computed as $c=10^b$, yielding values uniformly distributed on a logarithmic scale over the range [0.1, 10]. This logarithmic mapping ensures that approximately equal numbers of samples fall within the intervals [0.1, 1] and [1, 10], thereby ensuring uniform sampling across orders of magnitude.
